# Angiogenic and angiostatic factors present in the saliva of malaria patients

**DOI:** 10.1186/s12936-022-04221-7

**Published:** 2022-07-14

**Authors:** Cecilia Elorm Lekpor, Felix Botchway, Kwadwo Asamoah Kusi, Andrew A. Adjei, Michael D. Wilson, Jonathan K. Stiles, Nana O. Wilson

**Affiliations:** 1 Department of Pathology, Korle-Bu Teaching Hospital, University of Ghana Medical School, Accra, Ghana; 2 Department of Chemical Pathology, University of Ghana Medical School, Korle-Bu Teaching Hospital, Accra, Ghana; 3 Department of Immunology, Noguchi Memorial Institute for Medical Research, University of Ghana, Accra, Ghana; 4 Department of Parasitology, Noguchi Memorial Institute for Medical Research, University of Ghana, Accra, Ghana; 5 Department of Microbiology, Biochemistry & Immunology, Morehouse School of Medicine, Atlanta, GA USA

**Keywords:** Malaria, Malaria diagnosis, Saliva, Angiogenic, Angiostatic factors, Angiopoietin-1, Angiopoietin-2, CXCL10

## Abstract

**Background:**

Malaria related mortality is associated with significant deregulation of host inflammatory factors such as interferon-inducible protein 10, a member of the CXC or α-subfamily (CXCL10), and host angiogenic factors such as angiopoietin 1 (Ang-1) and angiopoietin 2 (Ang-2). However, detection of these factors in malaria patients requires the drawing of blood, which is invasive and increases the risk of accidental blood-borne infections. There has been an increased interest in the use of saliva as the body fluid of choice for the diagnosis of many infectious diseases including malaria. Here, saliva levels of CXCL10, Ang-1, and Ang-2 previously shown to be predictive of severe malaria in malaria patients in Ghana were assessed in malaria patients.

**Methods:**

This study was conducted in the Shai-Osudoku District Hospital in Dodowa, Accra, Ghana and the study population comprised 119 malaria patients and 94 non-malaria subjects. The non-malaria subjects are healthy community participants with no malaria infection. Plasma and saliva levels of CXCL10, Ang-1 and Ang-2 of the study participants were measured using an enzyme-linked immunoassay. Complete blood counts of each participant were measured with a haematology autoanalyzer. Pearson correlation was used to evaluate the correlation between plasma and saliva levels of each biomarker in malaria patients. A p-value of < 0.05 was considered significant. Box plots of median biomarker concentrations were plotted. SPSS version 14.2 software was used for statistical analysis.

**Results:**

The non-malaria subjects had a median age of 29 years compared to 23 years for malaria patients (p = 0.001). Among the malaria patients, there was a strong significant relationship between CXCL10 (R^2^ = 0.7, p < 0.0001) and Ang-1 (R^2^ = 0.7, p < 0.0001). Malaria patients had lower saliva levels of Ang-1 (p = 0.009) and higher saliva levels of CXCL10 (p = 0.004) and Ang-2 (p = 0.001) compared to non-malaria subjects.

**Conclusions:**

This study provides the first evidence of elevated levels of CXCL10 and Ang-2 in the saliva of malaria patients. Detection of CXCL10, Ang-1 and Ang-2 in saliva may have a potential application for non-invasive malaria diagnosis.

## Background

Malaria remains a major public health problem with an estimated 229 million cases and 409,000 related deaths globally in 2019, with 64% of the deaths occurring among children aged under 5 years [1]. Rapid and accurate diagnosis of malaria is key to effective control and management of the disease and avoids unnecessary presumptive treatment [2] as well as misuse and abuse of malaria treatment [3].

Microscopic examination has been the mainstay of malaria diagnosis. However, depending on the microscopist the sensitivity and specificity under field conditions may be as low as 10% and 71%, respectively [4]. In addition, microscopy is laborious and species determination at low parasitaemia densities is challenging [4, 5]. To overcome the limitations of microscopy, development of accurate, sensitive, and cost-effective rapid diagnostic tests (RDTs) that detect *Plasmodium* antigen in blood was promoted [6]. However, malaria cases sometimes go undetected using RDTs due to their inaccurate use, poor storage conditions and failure to detect low parasitaemia and this could result in the continuous transmission of malaria [7]. Furthermore, RDTs rely on finger-prick blood sampling which requires trained personnel, poses risk of infection and can complicate cooperation in children and communities with blood taboos [8]. Thus, there is an urgent need for the development of a simple non-invasive, cost-effective diagnostic tool that reduces the need for blood collection and is applicable to malaria-endemic and resource-limited areas.

Recently, saliva has become a viable alternative body fluid for analytical purposes and experimentation due to its ease of use and access by healthcare staff with limited training and minimal need for specialized equipment use [5]. Biomarkers have become very useful for the diagnosis of diseases such as cancer, diabetes, autoimmune diseases, and HIV/AIDS and prediction of disease severity [10]. Some molecular markers of malignancy have been detected in saliva of patients with oral carcinomas and saliva-based diagnostic kits have been developed for the diagnosis of HIV and human papillomavirus [11, 12]. Biomarkers in blood have been used to assess patterns of inflammatory response to malaria and their association with the severity of the infection [13]. However, screening of unique specific and sensitive biomarkers in saliva to diagnose and predict severity of malaria has received limited attention and acceptability among malaria field researchers.

Angiopoietins are distinct members of a family of vascular growth factors which play important roles in mediating inflammation and quiescence within the vascular endothelium [14]. Plasma or serum angiopoietins have been studied extensively in other disease conditions such as inflammatory bowel disease [15] neoplastic, inflammatory, infectious pathologies, neuroendocrine tumours, rheumatoid arthritis and malaria [16–19]. CXCL10 is a potent angiostatic and proinflammatory chemokine induced by IFN-γ, TNF, and other factors and has chemotactic activity for activated Th1 lymphocytes which have been demonstrated to be prognostic biomarkers in severe malaria [20]. Previous studies in both Indian and Ghanaian malaria patients demonstrated elevated CXCL10 levels in serum and cerebrospinal fluid samples and these levels were found to be associated with an increased risk of fatal *Plasmodium falciparum*-mediated severe malaria [20–24]. To date, there has been no report or study on the detection and comparative analysis of CXCL10, Angiopoietin-1 (Ang-1), and Angiopoietin-2 (Ang-2) in saliva and blood of malaria patients.

In this study, the levels of CXCL10, Ang-1, and Ang-2 in saliva and plasma were evaluated and compared to the levels in malaria patients with non-malaria subjects (healthy community participants with no malaria infection) in Ghana with the goal of assessing their use for non-invasive diagnosis of the disease.

## Methods

### Study population

The study was a case–control study performed between March and June 2017 in the Shai-Osudoku District (SOD) Hospital in Dodowa, Accra Ghana. Malaria patients and non-malaria subjects were recruited from SOD Hospital. The study recruited 213 participants including 119 malaria patients and 94 non-malaria subjects. A structured questionnaire was used to collect relevant socio-demographic and clinical data. Participation in the study was voluntary, with an informed consent form signed by the patient, or in the case of children, by the parents/ guardians.

The study protocol was approved by the Ethical and Protocol Review Committee of the College of Health Sciences, University of Ghana. In this case–control study, the presence of Ang-1, Ang-2 and CXCL10 biomarkers levels in saliva of malaria patients were evaluated. Malaria case was defined as participants having acute febrile illness as defined by an axillary temperature ≥ 37.5 °C at presentation or history of fever within the last 72 h of *Plasmodium* infection. Malaria was confirmed as positive thick and thin blood film, RDT positive, plus parasitaemia ≥ 2500/μl in the blood. The controls were healthy community participants with negative RDT for pLDH/HRP-2 and have the absence of *P. falciparum* parasitaemia on a thick and thin peripheral blood-stained smear. These healthy community participants were classified as non-malaria subjects in this study. The non-malaria subjects also had no history of fever, temperature, known infectious diseases or meningitis and showed no signs of impaired consciousness. The non-malaria subjects were recruited from the communities in which the patients with malaria reside. Patients with conditions which affect plasma Ang-1, Ang-2, and CXCL10 levels, such as diabetes mellitus, hypertension, coronary artery disease, chronic inflammatory, and autoimmune diseases as well as cancer [23] were excluded from the study.

### Blood sampling and laboratory processing

Four millilitres (4 ml) of venous blood from each study participant was collected into ethylene diamine tetraacetic acid (EDTA) tubes. An aliquot of 2.0 ml of the blood sample was put into CPT and centrifuged for 15 min at 1000×*g* using the Forma 3L Gp 4500R centrifuge (Thermo electron corporation, MA, USA) to separate plasma, red cells and peripheral blood mononuclear cells (PBMCs) and the processed plasma was stored at − 80 °C. A complete blood count (CBC) was done with an Automated Hematological Analyzer (Mindray BC5300, China). Thick and thin blood film slides were prepared with the EDTA blood sample within 2 h of collection, Giemsa-stained and examined for parasitaemia. About 1.5 ml of unstimulated saliva was collected from each participant into an OMNIgene-Oral tube (DNA genoTek, Canada) containing preservatives. The sample was stored at 28–30 °C prior to analysis.

### Assays of Angiopoietin-1, Angiopoietin-2, and CXCL10

The plasma and saliva levels of CXCL10, Angiopoietin-1, and Angiopoietin-2 of each participant were measured using commercial Sandwich enzyme-linked-immunosorbent assays (R&D Systems, USA) according to the manufacturer’s instructions. The optical density was determined within 30 min using Spectra Max 190 fluorescence microplate reader (Molecular Devices Corp., Sunnyvale, CA) with the wavelength set to 540 nm.

### Statistical analysis

The data was entered in Statistical Package for the Social Sciences (SPSS) version 14.2 software (SPSS Inc., Chicago, Illinois, USA). Frequency tables generated for categorical demographic characteristics and anaemia status are summarized as percentages. The results for continuous variables are expressed as median with the interquartile range (IQR). Chi-square tests were used to examine the differences in categorical variables. Fisher’s exact test was used when cell count was < 5. The Kruskal Wallis test or Mann–Whitney U test was used to compare differences in median values continuous variables among malaria patients and non-malaria subjects. Pearson correlation was used to evaluate the correlation between plasma and saliva levels of each biomarker in malaria patients. A p-value of < 0.05 was considered significant. Box plots of median biomarker concentrations with 25th and 75th percentiles were plotted. SPSS version 14.2 software (SPSS Inc., Chicago, Illinois, USA) was used for statistical analysis. GraphPad Prism version 6 (La Jolla, CA) for windows was used to generate all the graphs.

## Results

### Study participants

A total of 213 participants were enrolled in the study which included 94 non-malaria subjects and 119 malaria patients (Table 1). There was no significant difference in gender distribution between malaria patients and non-malaria participants (Table 1). The median age for malaria patients was 23 years and that of non-malaria subjects was 29 years (p = 0.001) (Table 1).

**Table 1:** Demographic characteristics and complete blood count by malaria status

Characteristics	Non-Malaria (N = 94)	Malaria (N = 119)	p-value
Gender			
Male	27 (28.7%)	44 (37.0%)	0.205
Female	67 (71.3%)	75 (63.0%)	
Age (years)			
Median (IQR)	29 (22–35)	23 (17–31)	**0.001**
Blood count—median (IQR)			
White blood cells (× 10^3^/uL)	6.3 (4.8–7.3)	5.9 (4.6–7.5)	0.396
Red blood cells (× 10^6^/uL)	4.7 (4.2–5.0)	4.5 (4.0–5.0)	0.125
Red cell distribution width—standard deviation (fL)	42.9 (40.3–45.9)	43.5 (41.8–45.5)	0.390
Red cell distribution width—coefficient of variation (%)	14.2 (13.5–15.5)	14.7 (13.4–16.0)	0.238
Mean corpuscular volume (fL)	81.3 (76.0–85.2)	81.8 (76.0–85.4)	0.987
Haemoglobin (g/dL)	12.0 (11.2–13.5)	12.2 (10.5–13.5)	0.462
Mean corpuscular haemoglobin (pg)	27.1 (25.0–28.6)	27.7 (25.4–29.0)	0.211
Haematocrit (%)	36.9 (34.1–40.0)	36.4 (31.8–40.5)	0.169
Mean corpuscular haemoglobin concentration (g/dL)	33.2 (31.8–34.0)	33.6 (32.4–34.7)	0.211
Platelets (× 10^3^/uL)	232.5 (181.5–279.3)	152.0 (109.0–213.0)	< **0.001**
Platelet distribution width (fL)	11.4 (10.5–13.5)	12.9 (11.7–14.1)	< **0.001**
Mean platelet volume (fL)	9.4 (8.8–10.5)	9.8 (9.1–10.5)	0.103
Platelet larger cell ratio (%)	20.6 (17.1–29.3)	25.5 (20.5–30.0)	**0.006**
Lymphocytes (× 10^3^/uL)	2.1 (1.7–2.5)	1.2 (0.8–1.8)	< **0.001**
Neutrophils (× 10^3^/uL)	3.2 (2.1–4.5)	3.9 (2.6–5.3)	**0.040**
Anemia status			
Anaemia	15 (16%)	37 (31.1%)	**0.011**
Severe anaemia	1 (1.1%)	3 (2.5%)	0.632

For categorical variables, Chi-square test were used to compare for statistical differences between malaria patients and non-malaria study participants. For instances in which there were too few subjects per cell for the Chi-square test to be used, Fisher’s exact test was used to compare discrete outcomes and continuous variables were compared using Mann–Whitney tests. Values were reported as number of observations and percentage (%) for categorical variables or median and Interquartile Range (IQR) for continuous variables. Statistical significance was set at p < 0.05

### Complete blood count of participants

The median platelet count was significantly lower in malaria patients than in non-malaria subjects (p < 0.001) (Table 1). There was a significant difference in the absolute lymphocyte counts between the malaria patients and non-malaria subjects (p < 0.001) (Table 1). There was no significant difference in white blood cell count between malaria patients and non-malaria subjects (p = 0.396) (Table 1). Approximately 31% of the malaria patients were anaemic compared to 16% of non-malaria subjects (p = 0.011) (Table 1).

### Correlation of angiopoietin- (Ang-1), angiopoietin-2 (Ang-2), and CXCL10 between saliva and plasma sample among malaria patients

**Fig. 1:**
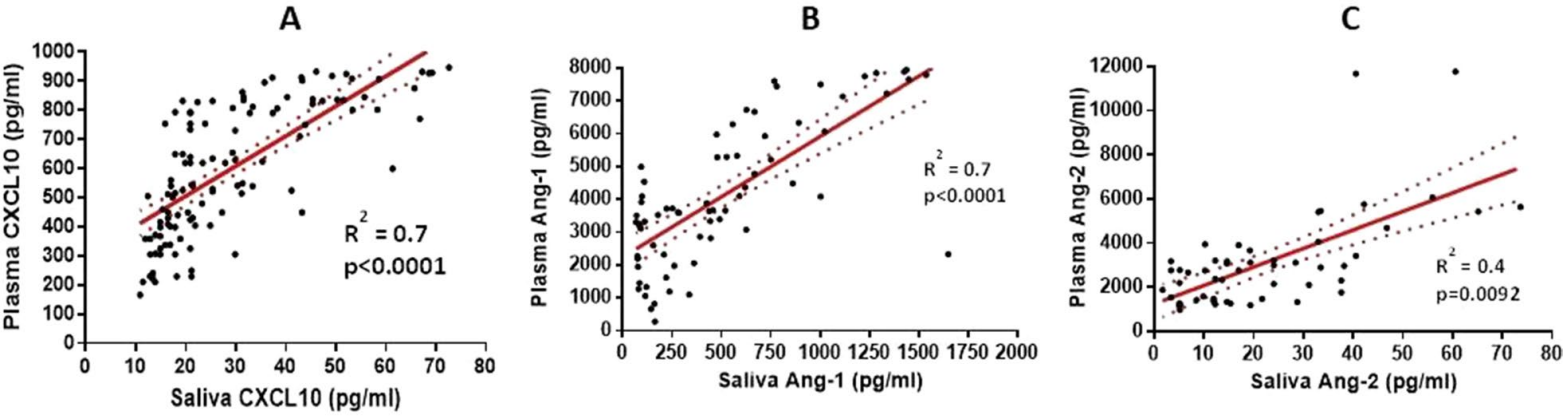
Relationship between saliva and plasma levels of angiopoietin-1, angiopoietin-2 and CXCL10 among malaria patients. **A** Correlation between saliva and plasma levels of CXCL10 among malaria patients. **B** Correlation between saliva and plasma levels of angiopoietin-1 among malaria patients. **C** Correlation between saliva and plasma levels of angiopoietin-2 among malaria patients

To determine the relationship of Ang-1, Ang-2 and CXCL10 between saliva and plasma, we performed a Pearson correlation to determine the association of these markers in saliva and plasma samples. There was a strong significant relationship in CXCL10 (R^2^ = 0.7, p < 0.0001) and Ang-1 (R^2^ = 0.7, p < 0.0001) levels between saliva and plasma (Fig. 1A and 1B). Although the relationship in Ang-2 levels between saliva and plasma was significant (P = 0.0092), the correlation was moderate (R^2^ = 0.4) (Fig. 1C).

### Plasma and saliva angiopoietin-1 (Ang-1), angiopoietin-2 (Ang-2), and CXCL10 levels by malaria status

**Fig. 2:**
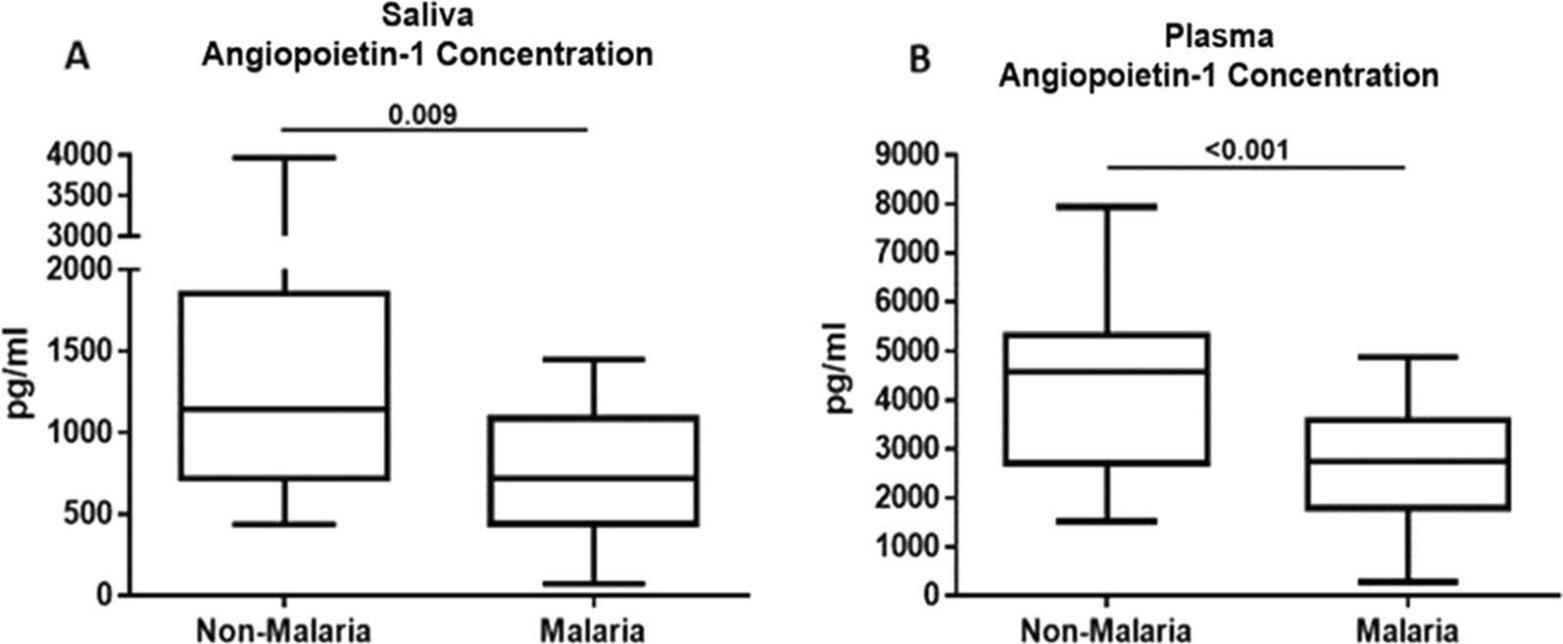
Saliva and plasma levels of angiopoietin-1 protein among the study participants. **A** Association of saliva angiopoietin-1 protein levels with malaria infection. Malaria patients have significantly lower saliva angiopoietin-1 levels than non-malaria subjects (p = 0.009). **B** Association of plasma angiopoietin-1 protein levels with malaria infection. Malaria patients have significantly lower plasma angiopoietin-1 levels than non- malaria subjects (p < 0.001). Box plot represent medians with 25th and 75th percentiles, bars for 10th and 90th percentiles. Significant differences of angiopoietin-1 median levels between the groups were determined by Mann–Whitney U test or Kruskal Wallis tests

**Fig. 3:**
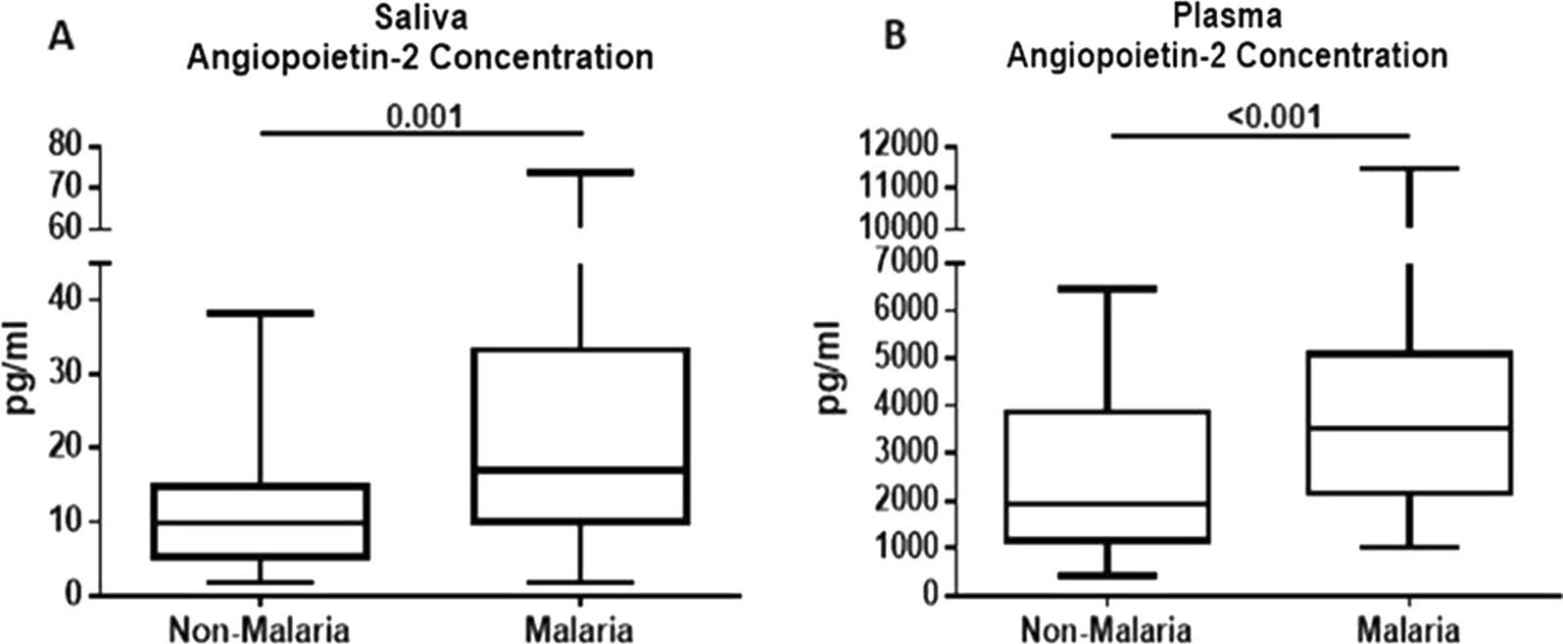
Saliva and plasma levels of angiopoietin-2 protein among the study participants. **A** Association of saliva angiopoietin-2 protein levels with malaria infection. Malaria patients have significantly higher saliva angiopoietin-2 levels than non-malaria subjects (p = 0.001). **B** Association of plasma angiopoietin-2 protein levels with malaria infection. Malaria patients have significantly higher plasma angiopoietin-2 levels than non-malaria subjects (p < 0.001). Box plot represent medians with 25th and 75th percentiles, bars for 10th and 90th percentiles. Significant differences of angiopoietin-2 median levels between the groups were determined by Mann–Whitney U test or Kruskal Wallis tests

**Fig. 4:**
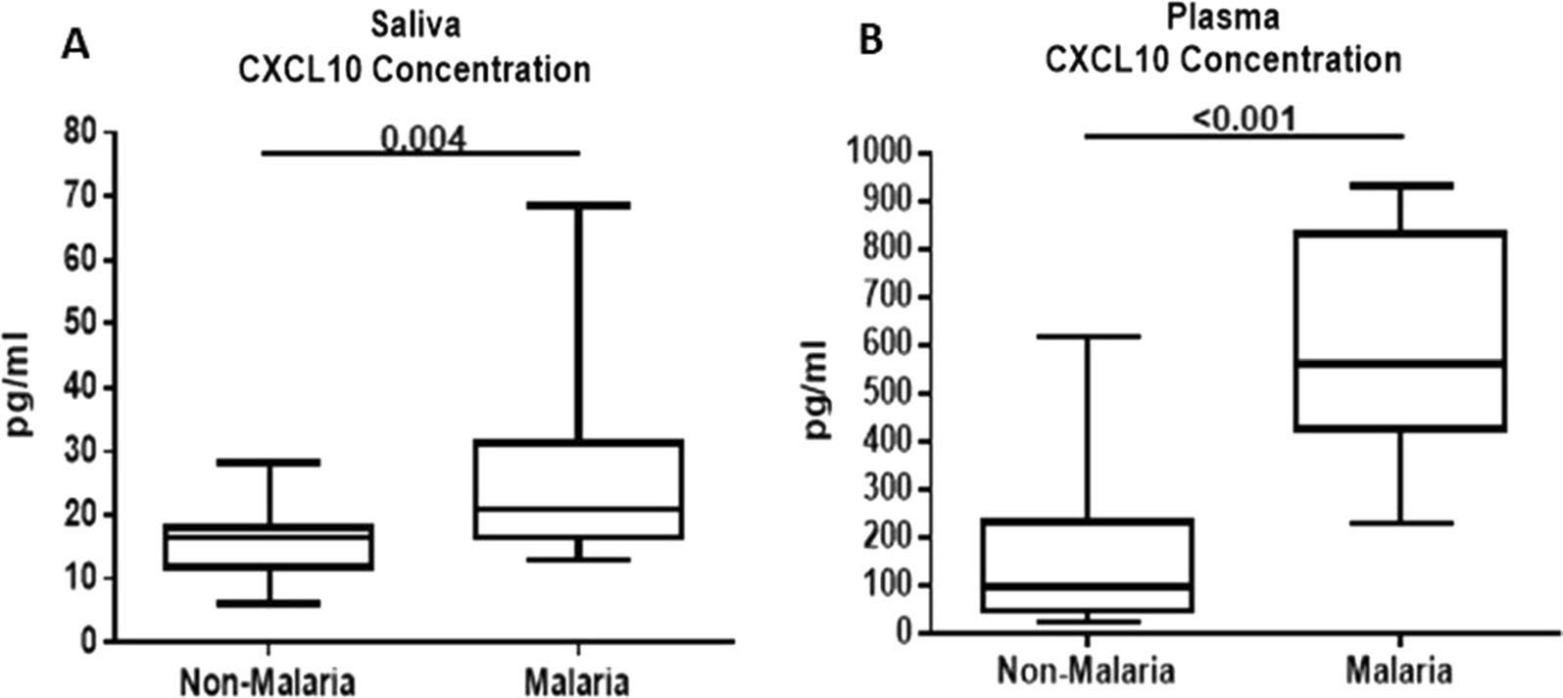
Saliva and plasma levels of CXCL10 protein among the study participants. **A** Association of saliva CXCL10 protein levels with malaria infection. Malaria patients have significantly higher saliva CXCL10 levels than non-malaria subjects (p = 0.004). **B** Association of plasma CXCL10 protein levels with malaria infection. Malaria patients have significantly higher plasma CXCL10 levels than non-malaria subjects (p < 0.001). Box plot represent medians with 25th and 75th percentiles, bars for 10th and 90th percentiles. Significant differences of CXCL10 median levels between the groups were determined by Mann–Whitney U test or Kruskal Wallis tests

Saliva Ang-1 levels were significantly lower in malaria patients than in non-malaria subjects, p = 0.009 (Fig. 2A). Similar patterns were observed in plasma Ang-1 levels where malaria patients had significantly lower levels compared to non-malaria subjects, p < 0.001 (Fig. 2B). Saliva Ang-2 levels were significantly higher in malaria patients than in non-malaria subjects (p = 0.001) (Fig. 3A). Similarly, plasma Ang-2 levels were significantly higher in malaria patients than in non-malaria subjects (p < 0.001) (Fig. 3B). CXCL10 levels were higher in both saliva (p = 0.004) and plasma (p < 0.001) when comparing malaria patients to the non-malaria subjects (Fig. 4A, B).

## Discussion

Differences in several haematological factors among malaria and non-malaria study participants were observed in this study. Consistent with findings from other studies, there was a significant difference in haemoglobin levels among malaria patients compared to the non-malaria group (Table 1). This could be due to hemolysis resulting from parasite infection and the elimination of parasitized red blood cells which could lead to anaemia [34]. However, there was no significant difference in severe anaemia in the malaria-infected group. This could be the result of older participants recruited in this study who may have better nutrition intake, stronger immunity and often develop partial immunity from previous malaria infection [9, 35]. Anaemia is one of the most common complications associated with malaria, especially in children and pregnant women who are susceptible to infection. There was also a significant decrease in platelet levels among the parasite-infected group compared to the uninfected group, a result that is consistent with other studies [34, 35]. This present study also observed a significant difference in neutrophils and lymphocyte levels in malaria patients which is consistent with other findings [9]. Biomarkers such as Ang-2 and CXCL10 have been found to be elevated in plasma during malaria parasite infection [20–25]. Previous report has demonstrated an elevated Ang-2/Ang-1 ratio to be used to assess the severity of malaria [25]. In this study, plasma and saliva Ang-2 levels were significantly higher in malaria patients as compared to non-malaria subjects. This study also revealed significantly lower levels of Ang-1 in both plasma and saliva samples of malaria patients.

Previous studies have demonstrated low plasma levels of Ang-1 and high values of Ang-2 to be associated with severe *Plasmodium* infections [25]. During *P. falciparum* infection, the production of angiogenic factors has been found to be associated with an increase in the cytoadherence of infected erythrocytes to the vascular endothelium [26]. Ang-1 and Ang-2 are the main regulators of endothelial activation and integrity, and their levels have been found to be altered during inflammatory conditions due to endothelial activation [27, 28]. Other studies have also described Ang-1 and Ang-2 levels as reliable biomarkers in distinguishing uncomplicated malaria from severe malaria [29]. This study only focused on determining the levels of these markers in saliva in malaria patients compared to non-malaria subjects. However, other studies have described Ang-1 and Ang-2 levels in relation to severe malaria, where levels of Ang-1 were considered the best discriminator of uncomplicated malaria from severe malaria and were found to serve as a reliable diagnostic biomarker [24, 29].

In line with this study, Sahu et al. [30] reported elevated levels of plasma Ang-2 and lower plasma Ang-1 levels in malaria patients and found Ang-2 to be a better prognostic marker to signal any imminent severity in *P. falciparum* malaria infection than other markers of disease severity. Meanwhile, a similar study also reported an increase in plasma Ang-2 levels in adults and children with severe malaria and found Ang-2 to be a better predictor of death than other markers of disease severity [18].

Elevated Ang-2 levels have also been associated with endothelial damage [26, 31]. Although lower levels of Ang-1 have been found in malaria patients, higher levels were detected in patients who have been treated and recovered from falciparum malaria, suggesting that Ang-1 may be involved in the pathogenesis of malaria [4, 32, 33]. Although the levels of these markers in saliva were not as high as in plasma in the current study, it is noteworthy that Ang-1, Ang-2 and CXCL10 were all detectable in the saliva of malaria patients. Further, there was a correlation between saliva and plasma levels of Ang-1, Ang-2 and CXCL10 among malaria patients suggesting any increase or decrease in these biomarkers in plasma was also observed in saliva. Currently, there is a lack of a liable diagnostic tool for the identification of severe malaria. However, these markers have been shown to be discriminators of uncomplicated malaria and can serve as a reliable diagnostic marker [24, 29]. Detection of these markers in saliva offers a practical alternative for malaria diagnosis in the case of severe malaria where these markers have been shown to discriminate between uncomplicated malaria and severe malaria. Detection of these markers in saliva offers some distinct advantages over blood; a collection of saliva samples is non-invasive compared to blood and painless to collect.

This study provides the first evidence of Ang-1, Ang-2, and CXCL10 in saliva discriminating between malaria patients and non-malaria subjects. With the future goal of incorporating these factors in point-of-care diagnostic kits for malaria detection, the discovery of these markers in the saliva of malaria patients could allow for accurate and early detection of malaria for immediate intervention. Furthermore, this will provide a non-invasive approach that will facilitate and make for easy screening of large populations in epidemiological surveys in endemic countries.

## Limitations

The small number of patients examined restricted the possibilities of extrapolating the results to other populations and will require further studies to enable firm conclusions to be drawn. Additionally, the subjects in the study were not tested for other infections that have been associated with these biomarkers to determine if these biomarkers will also differentiate malaria parasite infection from other infections associated with these biomarkers. Therefore, further studies need to be conducted to test for the differentiation of these biomarkers between malaria and other infections.

## Conclusion

This study indicate that key biomarkers of malaria, Ang-1, Ang-2, and CXCL10, could be detected in saliva and may have potential application for clinical diagnosis of malaria. After further optimization of the assay protocol to improve sensitivity, it could serve as a supplement or alternative to blood sampling for testing malaria in malaria-endemic areas and large-scale epidemiology surveys. Further studies in different settings or populations as well as the sensitivity and specificity of these biomarkers in saliva are however needed to validate our current findings.

## Data Availability

Data is available upon request.
